# Expression of migration-related genes is progressively upregulated in murine Lineage^-^Sca-1^+^c-Kit^+ ^population from the fetal to adult stages of development

**DOI:** 10.1186/scrt14

**Published:** 2010-05-20

**Authors:** Jesús Ciriza, Marcos E García-Ojeda

**Affiliations:** 1 University of California, Merced, School of Natural Sciences, 5200 North Lake Road, Merced, CA 95343, USA

## Abstract

**Introduction:**

Hematopoietic stem cells (HSCs) follow a genetically programmed pattern of migration during development. Extracellular matrix and adhesion molecules, as well as chemokines and their receptors, are important in adult HSC migration. However, little is known about the role these molecules play at earlier developmental stages.

**Methods:**

We have analyzed by quantitative polymerase chain reaction (qPCR) array the expression pattern of extracellular matrix and adhesion molecules as well as chemokines and chemokine receptors in Lineage^-^Sca-1^+^c-Kit^+ ^(LSK) cells at different stages of development, in order to characterize the role played by these molecules in LSK. Data were represented by volcano plots to show the differences in expression pattern at the time points studied.

**Results:**

Our results show marked changes in the expression pattern of extracellular matrix, adhesion molecules, chemokines and their receptors with developmental age, particularly in later stages of development. Ten molecules were significantly increased among the LSK populations studied. Our screen identified the upregulation of *Col4a1*, as well as molecules involved in its degradation (*Mmp2, Timp2*), with development. Other genes identified were *Sell*, *Tgfbi*, and *Entpd1*. Furthermore, we show that the expression of the chemokines *Ccl4*, *Ccl9*, *Il18 *and the chemokine receptor *Cxcr4 *increases in LSK cells during development.

**Conclusions:**

Several genes are upregulated in the LSK population in their transition to the bone marrow microenvironment, increasing at later stages of development. This gene pattern should be emulated by embryonic stem cell-derived hematopoietic progenitors in order to improve their properties for clinical applications such as engraftment.

## Introduction

Hematopoietic stem cells (HSCs) are the best characterized stem cell population in adult organisms. They differentiate into all blood lineages [1] and self-renew to keep a constant pool of HSCs throughout life [2]. HSCs lack expression of mature lineage markers (Lin^-^) and have high expression of Stem cell antigen-1 (Sca-1) and c-Kit receptor tyrosine kinase (stem cell factor receptor) [1]. All these markers define the LSK population (Lin^-^Sca-1^+^c-Kit^+^), which contains a heterogeneous mixture of hematopoietic progenitors including long term HSCs (LT-HSCs), short term HSCs (ST-HSCs), and multipotent progenitors (MPPs) [1]. While ST-HSC are responsible for rapid reconstitution of myeloablated transplant recipients, LT-HSCs possess complete self-renewal properties, which are important in long term reconstitution applications, such as therapeutic bone marrow transplantation [3]. LT-HSC can be distinguished from ST-HSC and MPP by the expression of Signaling Lymphocyte Activation Molecule (SLAM) family markers [2]: only LT-HSCs express the SLAM family marker CD150 and lack expression of CD48 and CD41 [4]. SLAM markers have been shown to be useful in identifying adult as well as fetal LT-HSCs after day 14.5 of gestation (FL14.5) [5,6].

During embryonic development, HSCs follow a defined pattern of migration through different anatomical locations [6-8]. At early stages of development, HSCs have been found in both yolk sac and the para-aortic splanchnopleura (pSp) as well as in the aorta, gonad and mesonephros (AGM) region [9-12]. The fetal liver is populated with LT-HSCs by day 11.5 of gestation. However, although the fetal bone marrow is present by 15.5 days post coitum (dpc), LT-HSCs cannot be detected in this tissue until day 17.5 of gestation. Christensen et al. hypothesized that circulating LT-HSC, although chemotactic by 14.5 dpc to the bone marrow recruiting chemokine stromal cell derived factor-1α (SDF-1α), would not colonize the fetal bone marrow until a suitable microenvironment is present [8]. Alternatively, LT-HSCs circulating in fetal blood might not possess the appropriate chemokine receptor or adhesion molecule repertoire required for bone marrow homing and migration. The analysis of the expression of these molecules in LT-HSC and LSK populations could shed light into the mechanisms involved during the process of embryonic hematopoietic progenitor migration as well as in the physiological hematopoietic progenitor migration observed in adult organisms [13].

Extracellular matrix and adhesion molecules are necessary for the migration and homing of adult HSC into the bone marrow [14,15]. For example, β1 integrin deficient HSC cannot colonize the fetal liver, spleen and adult bone marrow [16,17] while antibody blocking [18] or conditional deletion [19] of α4 integrin results in reduced bone marrow homing of HSC. Likewise, blocking matrix metallopeptidase 9 (MMP9) activity in HSC by antibodies or genetic deletion impairs HSC motility [20]. Chemokines and their receptors have been also implicated in both mobilization and bone marrow homing of transplanted adult HSC [15]. SDF-1α, also called CXCL12, whose receptor CXCR4 is expressed by HSC, has chemoattractant properties to hematopoietic progenitor cells in humans and mice [21,22]. Moreover, mice deficient in SDF-1α or CXCR4 do not establish bone hematopoiesis but have normal fetal liver hematopoiesis [23-25]. Adult marrow LSK cells also express mRNA for the chemokines receptors *Ccr3 *and *Ccr9*, although they fail to migrate to the ligands for these receptors [22]. In the same study, the chemokines receptors *Xcr1*, *Cxcr2 *and *Cxcr5 *were detected in some but not all of the LSK cells tested, exemplifying the variation in the expression of these molecules among the LSK population. Altogether, an exhaustive expression analysis of extracellular matrix, adhesion, and chemokine molecules has not been done in LSK at different embryonic developmental stages or anatomical locations.

In this study, we investigated the expression of extracellular matrix and adhesion molecules, as well as chemokines and their receptors, in LSK populations at different developmental times by a qPCR array. We hypothesized that their expression will vary in LSK populations depending on developmental stage and anatomical location. We isolated LSK and LT-HSC cells at different stages of development by flow cytometry. Subsequently, we used qPCR arrays to examine the expression of extracellular matrix, adhesion, chemokines and chemokine receptor molecules. Our results show that LSKs from fetal bone marrow at 17.5 dpc share a molecular pattern similar to fetal liver LSKs at 17.5 dpc and adult bone marrow, whereas LSK cells in the fetal liver at 14.5 dpc have lower expression level of the molecules studied. Moreover, we observed that there is a significant progressive upregulation of genes involved in HSC migration (including the chemokine receptor *Cxcr4 *as well as molecules related to type IV collagen alpha1 synthesis, degradation and regulation (*Col4a1, Mmp2, Timp2*)) during development, with the highest expression in adult tissue. Taken together, our study is the first to directly compare the expression of adhesion molecules and chemokines in the LSK population during different stages of fetal development.

## Materials and methods

### Mice

C57BL/6 (B6) mice were purchased from The Jackson Laboratory (Bar Harbor, ME, USA) or bred in house and housed in sterile microisolator cages with sterile feed and autoclaved water. They were euthanized by CO_2 _asphyxiation. All procedures were approved by the UC Merced Institutional Animal Care and Use Committee (IACUC).

### Antibodies

The following monoclonal antibodies were purchased from BioLegend (San Diego, CA, USA) and used in flow cytometry: PE-Cy5-conjugated anti-CD3 (145-2C11), anti-CD4 (RM4-5), anti-CD8 (53-6.7), anti-CD11b (M1/70), anti-CD19 (6D5), anti-NK1.1 (PK136), anti-Ter119 (Ter119) and anti-GR1 (RB6-8C5), PE-conjugated anti-Sca-1 (E13-161.7), APC-conjugated anti-c-Kit (2B8), PE-Cy7-conjugated anti-CD150 (TC15-12F12.2) and FITC-conjugated anti-CD48 (HM48-1). FITC-conjugated anti CD41 (MWReg30) and purified anti-CD16/32 (2.4G2) were purchased from eBioscience (San Diego, CA, USA). Each antibody was carefully titrated and used at a concentration that gave the highest signal with the lowest background, following staining of control bone marrow cells.

### Tissue isolation

Breeder mice were mated in the early evening and were checked for vaginal plugs the following morning. The morning on which vaginal plugs were observed was designated 0.5 dpc. Fetal liver and limbs were dissected from fetuses at 14.5 and 17.5 dpc and placed in cold M199+ media (Invitrogen, Carlsbad, CA, USA) with 2% FBS (Atlanta Biologicals, Lawrenceville, GA, USA). Single-cell suspensions were prepared by triturating the tissues through a 70 μm nylon mesh screen. At least three pregnant females were used for each time point. Those fetuses that appeared developmentally advanced or delayed in any age group were discarded. Adult bone marrow obtained from the hind leg bones of four-week old mice was flushed with a 25 G needle in cold M199+ media with 2% FBS. Single cell suspension was filtered through a 70 μm nylon cell strainer.

### Cell staining for flow cytometric sorting and analysis

Single cell suspensions from the different tissues were stained with anti-CD16/32 antibody to block FcγII/III receptors. Fc blockage was followed by staining with an antibody cocktail containing PE-Cy5-conjugated anti-CD3, anti-CD4, anti-CD8, anti-CD11b, anti-CD19, anti-NK1.1, anti-Ter119 and anti-GR1 (Lineage markers), followed by staining with anti-PE microbeads (Miltenyi Biotec, Auburn, CA, USA). The Lin^+ ^cells were depleted using the AutoMACS ("Deplete_S" protocol) to enrich the lineage negative fraction containing hematopoietic progenitors. Subsequently, this fraction was stained with PE-conjugated anti-Sca-1, APC-conjugated anti-c-Kit, PE-Cy7-conjugated anti-CD150, as well as FITC-conjugated anti-CD41 and anti-CD48. Propidium iodide (0.5 μg/ml final concentration) was added before sorting to exclude dead cells. LSK cells were isolated using a FACSAria™ flow cytometer controlled by BD FACSDiva™ software (version 4.1, Becton Dickinson, Franklin Lakes, NJ, USA). Unstained cells were used to evaluate autofluorescence. Each experiment was carefully compensated by staining BD™ CompBeads (BD Biosciences, San Jose, CA, USA) with the appropriate antibodies and using BD FACSDiva™ software.

### RNA isolation

RNA was extracted from sorted LSK cells using TRIzol^® ^reagent (Invitrogen), following the manufacturer's instructions. Following DNAse I (Roche, San Francisco, CA, USA) treatment, the concentration of RNA was determined using a Nanodrop spectrophotometer (Thermo Scientific, Waltham, MA, USA).

### Quantitative PCR array

Two μg of RNA were processed with RT^2 ^First Strand Kit (SA Biosciences, Frederick, MD, USA) according to the manufacturer's specifications. Quantitative PCR analysis of extracellular matrix and adhesion molecules, as well as for chemokines and receptors, were assessed with the Extracellular Matrix and Adhesion Molecules PCR Array (SA Biosciences) [26], the Chemokines & Receptors PCR Array (SA Biosciences) [27], RT^2 ^SYBR^® ^Green qPCR master mix (SA Biosciences) and a Stratagene Mx3000p qPCR System (Santa Clara, CA, USA). At least three RNA samples per time point were analyzed.

### Data analysis

Flow cytometry data analysis was performed using FlowJo software (version 7.2.4, Tree Star Inc., Ashland, OR, USA). Tukey and Student *t*-test statistical analysis were performed with SPSS software (version 13.0, IBM, Chicago, IL, USA). PCR array data were analyzed by the RT^2 ^Profiler PCR Array Data Analysis web portal from SA Biosciences based in the ΔΔCt method with five different housekeeping genes and represented by volcano plots [28].

## Results

### Analysis of the frequency of LSK and LT-HSCs in different microenvironments during development

**Figure 1:**
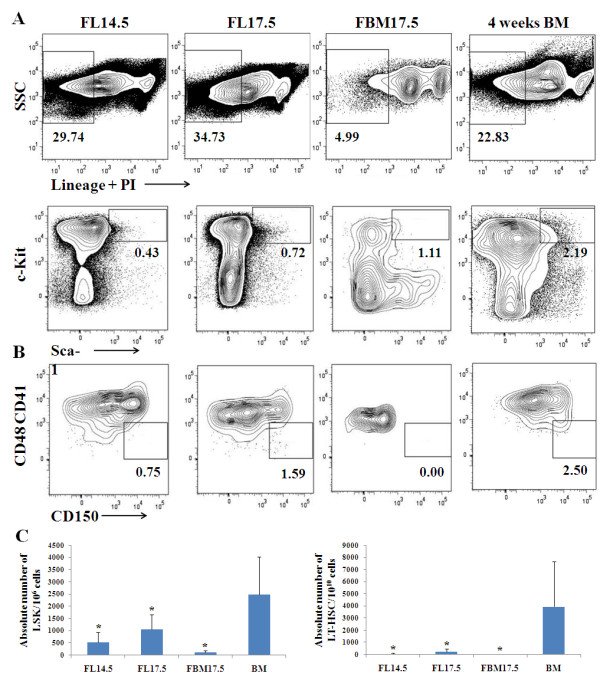
**Frequency analysis of hematopoietic stem cells**. **(a) **Live, lineage negative-to-low cells (gated cells in upper panels) were analyzed for the expression of c-Kit and Sca-1 (lower panels) at different microenvironments and stages of development. Gate shows the Lineage^-/low^Sca-1^+^c-Kit^+ ^(LSK) population, containing hematopoietic stem cells (HSC). **(b) **Analysis of LSK population for the expression of CD150 versus CD41 and CD48 in different microenvironments and stages of development. Gate shows LSK CD150^+^CD41^-^CD48^- ^LT-HSC. Data shown in 5% of contour plots, using Bi-Exponential scaling. **(c) **Representation of the absolute number of LSK and LT-HSC in the different microenvironment and stages of development studied. Note: * = *P *< 0.05, obtained by Tukey analysis, indicating that values are significant when compared to the adult bone marrow.

We analyzed the frequency of the LSK population in lineage depleted fetal livers at 14.5 dpc (FL14.5) and 17.5 dpc (FL17.5), in fetal bone marrow at 17.5 dpc (FBM17.5) and in bone marrow from four-week old adult (adult BM) mice (Figure 1a). The average percentage of LSK cells detected in FL14.5 was 0.45 ± 0.26%, 0.78 ± 0.34% in FL17.5, 1.9 ± 0.57% in FBM17.5 and 2.94 ± 0.7% in adult BM (Table 1). The percentage of adult BM LSK cells is in agreement with previously published work [29], and validates our LSK gating strategy (Figure 1a). These data show that the frequency of LSK cells increases during development. Note that by 17.5 dpc the fetal bone marrow has a higher frequency of LSK cells than the fetal liver. By means of Tukey and Student's *t*-test statistical analysis, we established that the difference between the frequencies of LSK cells in the adult bone marrow versus the fetal liver at different stages of development is statistically significant (*P *< 0.05). Furthermore, there was a statistically significant difference between the percentage of LSK cells in the FL14.5 and FBM17.5 samples. In contrast, there was no significant difference between the frequency of LSK cells in adult BM or FL17.5 versus FBM17.5. When the absolute numbers of LSK cells were determined, all samples gave significant differences when compared to the adult bone marrow (Figure 1c, left panel).

**Table 1:** Frequency of LSK and long-term hematopoietic stem cells at different microenvironments and stages of development

Tissue	**% LSK**^+^	**% LSK**^+^**CD150**^+^**CD48**^-^**CD41**^-^
FL14.5 (n = 9)	0.45 ± 0.26 *†	0.004 ± 0.004 *
FL17.5 (n = 8)	0.78 ± 0.34 *	0.011 ± 0.006 *
FBM17.5 (n = 6)	1.19 ± 0.57	0.00 ± 0.00 *
Adult BM (n = 11)	2.94 ± 0.70	0.17 ± 0.08

Data shown as mean frequency of live, Lin^-/low ^gated events. The number of independent samples evaluated as n. An asterisk (*) indicates a significant difference (*P *< 0.05) of LSK or LT-HSCs percentage with adult bone marrow (BM) as reference sample (Tukey and Student's *t*-test analysis). A dagger (†) indicates a significant difference (*P *< 0.05) of LSK percentage with FBM17.5 as reference sample (Tukey and Student's *t*-test analysis). Adult BM, four-week old adult bone marrow; FBM17.5, fetal bone marrow at day 17.5 of gestation; LSK, Lineage^- ^Sca-1^+ ^c-Kit^+^; LT-HSC, Long Term-Hematopoietic Stem Cells.

As the LSK population contains both LT-HSCs and more developed progenitors, we analyzed the frequency of LT-HSCs defined by the LSK CD150^+^CD41^-^CD48^- ^phenotype [5] (Figure 1b). We analyzed LT-HSC based on the stringent LSK CD150^+^CD41^-^CD48^- ^gate shown by Weksberg and colleagues to harbor 86% of the LSK population [30]. In FL14.5, the mean frequency value of LT-HSC obtained was 0.004 ± 0.004%, whereas the FL17.5 had 0.001 ± 0.006% and adult BM had 0.17 ± 0.08% (Table 1). Few if any LT-HSCs were detected in the FBM17.5, reflecting the rare frequency of LT-HSC in this tissue at this developmental time. Tukey statistical analysis showed that the difference between the frequencies of cells in the adult BM with the rest of the microenvironments studied is significant (*P *< 0.05). However, no significant differences were found between the embryonic microenvironments when compared among them. When the absolute numbers of LT-HSC cells were determined, all samples gave significant differences when compared to the adult bone marrow (Figure 1c, right panel). Overall, the data indicate that there is a progressive expansion in the frequency and number of LSK as well as LT-HSCs during development.

### Quantification of extracellular matrix and adhesion molecules expressed in LSK cells in different microenvironments and stages of development

Extracellular matrix and adhesion molecules have been implicated in hematopoietic stem cell migration [31,32]. To determine if their expression changes in different microenvironments during development, we analyzed the expression of these molecules in the LSK population by quantitative PCR array. The Extracellular Matrix and Adhesion Molecules PCR Array (SA Biosciences) allows for the simultaneous analysis of 84 genes [26]. The array data were shown by volcano plots, which are one of the best mathematical representations available to compare the expression of multiple genes from two samples [33]. Volcano plots consider statistical significance (*P*-values) as well as fold change, organizing genes along dimensions of biological and statistical significance. Each dot in the plot represents a studied gene. The x-axis indicates biological impact (fold change) while the y-axis indicates the statistical reliability of the fold change.

**Figure 2:**
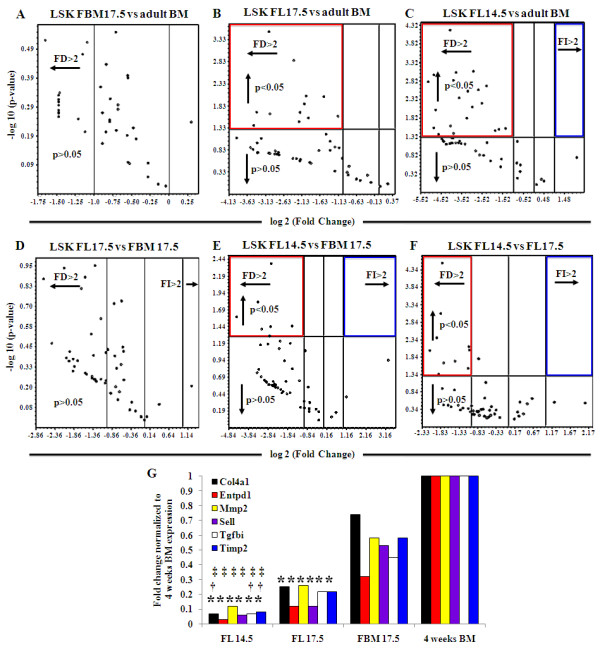
**Extracellular matrix and adhesion molecules gene expression in LSK from different microenvironments of development**. **(a-f) **Volcano plots comparing the extracellular matrix and adhesion molecules gene expression fold change between pairs of samples (reference sample indicated in the title of each plot). Each dot represents the expression of one gene. Dots above the horizontal line show significant difference (*P *< 0.05). Dots to the right of the vertical line show fold increase while dots on the left show fold decrease (above a value of two). FI, fold increase, FD, fold decrease. Central vertical line represents a fold value of zero. Red squares highlight significant fold decrease, while blue squares indicate significant fold increase. **(g) **Expression fold change of genes that showed significant difference between FL14.5, FL17.5, FBM17.5 and adult BM. Genes analyzed include *Col4a1, Entpd1, Mmp2, Sell, Tgfbi, and Timp2*. Gene expression was normalized to adult BM. Note: * = significant difference (*P *< 0.05) with BM; † = significant difference (*P *< 0.05) with FBM17.5; ‡ = significant difference (*P *< 0.05) with FL17.5.

When represented by volcano plot, the comparison of extracellular matrix and adhesion molecule gene expression between LSK cells from FBM17.5 versus adult BM did not display any significant difference (Figure 2a), indicating that their gene expression pattern is similar. Likewise, no significant differences were observed between the gene expression patterns of LSK cells from the FL17.5 versus FBM17.5 samples (Figure 2d). However, 12 extracellular matrix and adhesion molecule genes were significantly downregulated (fold decrease, FD >2) when the FL17.5 LSK population was compared to LSK cells in adult BM (Figure 2b). Taken together, our data suggest that the expression pattern of extracellular matrix and adhesion molecules in FBM17.5 LSKs is transitional between FL17.5 and adult bone marrow.

LSKs in FL14.5 showed significant differences in gene expression with the rest of the microenvironments studied (Figure 2c, e and 2f). Twenty genes are significantly downregulated (*P *< 0.05) when FL14.5 LSK cells are compared with adult BM LSK (Figure 2c), 7 genes when compared to FBM17.5 LSK (Figure 2e) and 10 genes when compared to FL17.5 LSK (Figure 2f). These data indicate that the larger the developmental distance between LSK populations, the more differences can be found in their expression of extracellular matrix and adhesion molecule genes.

Only 27 of the 84 extracellular matrix and adhesion molecule genes analyzed by qPCR array showed expression in the LSK population at all developmental points studied (Table 2). These 27 genes encompass the genes that show significant differences in all the volcano plots. These genes were grouped based in their function as: transmembrane molecules (thirteen genes), cell-cell adhesion molecules (one gene), other adhesion molecules (five genes), collagens and extracellular matrix structural constituents (two genes), extracellular matrix proteases (two genes), extracellular matrix protease inhibitors (one gene) and other extracellular matrix molecules (three genes). Of these 27 genes, only six (*Col4a1, Entpd1, Mmp2, Sell, Tgfbi *and *Timp2*) showed statistically significant differences among the various times of development (Figure 2g). These genes were upregulated in the adult BM LSK population when compared to the fetal liver samples regardless of developmental time. Although the expression of these six genes increased during development, no significant differences were found in their expression in LSK cells from FL17.5, FBM17.5 and adult BM. However, these genes were significantly upregulated in LSK cells from FL17.5 when compared to FL14.5. Only *Col4a1, Tgfbi *and *Timp2 *were significantly upregulated in FBM17.5 LSK when compared to FL14.5 LSK. These data suggest that these six extracellular matrix genes could be playing an important role in the LSK population during development, possibly in their migration between niches.

**Table 2:** Expression of extracellular matrix and adhesion molecules in LSK populations across different microenvironments and stages of development

Gene	FL14.5	FL17.5	FBM17.7	Adult BM
Transmembrane molecules				
*Cd44*	0.15	0.24	0.47	1
*Entpd1*	0.03	0.12	0.32	1
*Itga4*	0.38	0.49	0.62	1
*Itgal*	0.63	0.8	1.23	1
*Itgam*	0.3	0.43	0.72	1
*Itgav*	0.27	0.32	0.54	1
*Itgb1*	0.57	0.46	0.68	1
*Itgb2*	0.2	0.35	0.61	1
*Itgb3*	1.28	0.54	0.58	1
*Pecam1*	0.2	0.56	0.82	1
*Sell*	0.06	0.12	0.53	1
*Selp*	0.13	0.2	0.62	1
*Sgce*	0.1	0.39	0.68	1
Cell-cell adhesion molecules				
*Vcam1*	0.08	0.27	0.36	1
Other adhesion molecules				
*Ctnna1*	0.17	0.36	0.56	1
*Ctnnb1*	0.56	0.39	0.97	1
*Emilin1*	0.62	0.86	0.82	1
*Lamb2*	0.06	0.11	0.36	1
*Lamc1*	0.57	0.43	0.68	1
Collagens and extracellular matrix structural constituents				
*Col4a1*	0.07	0.25	0.74	1
*Col4a2*	0.11	0.46	0.68	1
Extracellular matrix proteases				
*Adamts5*	0.03	0.1	0.61	1
*Mmp2*	0.12	0.26	0.58	1
Extracellular matrix proteases				
*Timp2*	0.08	0.22	0.58	1
Other extracellular matrix molecules				
*Ecm1*	0.33	0.55	0.56	1
*Tgfbi*	0.07	0.22	0.45	1
*Thbs1*	4.26	1.02	0.43	1
Housekeeping gene				
*Hprt1*	0.13	0.18	0.44	1

Table demonstrates fold change values for samples in which gene expression was detected at all the points of study, compared to gene expression in adult bone marrow. Adult BM, four-week old adult bone marrow; FBM17.5, fetal bone marrow at Day 17.5 of gestation; FL14.5, fetal liver at Day 14.5 of gestation; FL17.5, fetal liver at Day 17.5 of gestation; LSK, Lineage^- ^Sca-1^+ ^c-Kit^+^.

### Quantification of chemokines and chemokine receptors on LSK cells in different microenvironments and stages of development

**Figure 3:**
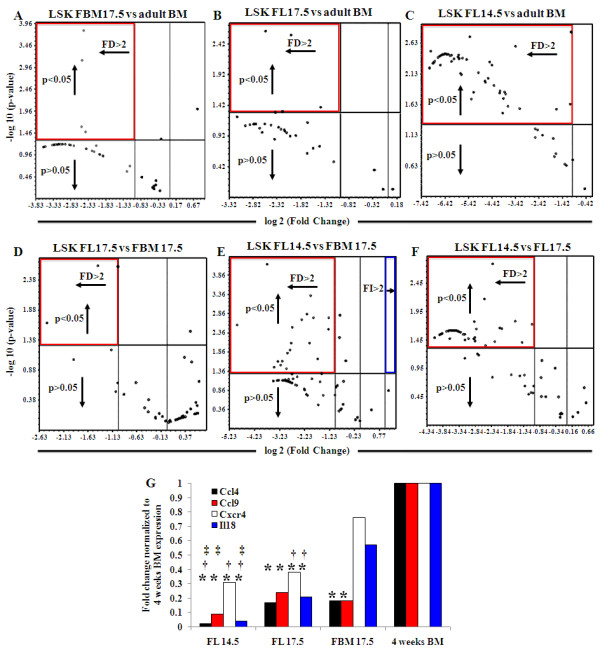
**Chemokines and receptors gene expression in LSK from different microenvironments of development**. **(a-f) **Volcano plots comparing chemokines and receptors gene expression fold change between pairs of samples (reference sample indicated in the title of each plot). Each dot represents the expression of one gene. Dots above the horizontal line show significant difference (*P *< 0.05). Dots to the right of the vertical line show fold increase while dots on the left show fold decrease (above a value of two). FI, fold increase, FD, fold decrease. Central vertical line represents a fold value of zero. Red squares highlight significant fold decrease, while blue squares indicate significant fold increase. **(g) **Expression fold change of genes that showed significant difference between FL14.5, FL17.5, FBM17.5 and adult BM. Genes analyzed include *Ccl4, Ccl9, Cxcr4 *and *Il18*. Gene expression was normalized to adult BM. Note: * = significant difference (*P *< 0.05) with BM; † = significant difference (*P *< 0.05) with FBM17.5; ‡ = significant difference (*P *< 0.05) with FL17.5.

Chemokines and their receptors play important roles in the migration of the HSC into the adult BM as well as during development [8,22]. We quantified and compared the gene expression pattern of these molecules in LSK populations from different microenvironments and stages of development. Sorted LSK cells from FL14.5, FL17.5, FBM17.5, and adult BM were analyzed by the Chemokine & Receptors PCR Array (SA Biosciences) for expression of 84 chemokine and chemokine receptor genes [27] and the data represented by volcano plots (Figure 3a-f). LSK cells from either FL17.5 or FBM17.5 showed a significant (*P *< 0.05) downregulation (FD >2) in four genes when compared with adult BM (Figure 3a, b). When the gene expression profile of FL14.5 and adult BM LSK were compared, 71 genes were significantly downregulated in FL14.5 (Figure 3c). Moreover, only three genes showed a significant downregulation in the FL17.5 LSK population compared to FBM17.5 LSK (Figure 3d). However, when FL14.5 samples were compared with FBM17.5, 19 genes showed a significant downregulation in the FL14.5 LSK population (Figure 3e). The Chemokine & Receptors PCR Array showed differences in the expression pattern between different microenvironments that were not observed with the Extracellular Matrix and Adhesion Molecules PCR Array. These data suggest that LSK cells in the fetal bone marrow microenvironment have unique characteristics. Lastly, when LSK cells from FL14.5 were compared to FL17.5 LSK, 57 genes were significant downregulated (Figure 3f). All but one of these 57 genes were included in the 71 genes differentially expressed between the FL14.5 and adult BM LSK, which indicates that the main change in the expression pattern of this group of genes happens in fetal development between 14.5 dpc and 17.5 dpc.

Among the 84 genes studied in the Chemokine & Receptors PCR Array, 17 genes were expressed in all the LSK populations examined (Table 3). These 17 genes encompass the genes that show significant differences in all the volcano plots, except the plots analyzing FL14.5 samples. They were grouped as: four chemokine (C-C motif) ligands, three chemokine (C-X-C motif) ligands, one chemokine (C-X-C motif) receptor and 10 other chemokines and related genes. However, only four genes had statistically significant differences between all the microenvironments studied: *Ccl4, Ccl9, Cxcr4 *and *Il18 *(Figure 3g). All these genes had significant differences when the adult BM LSK was compared with either fetal liver samples. Between adult BM and FBM17.5 LSK cells, only *Ccl4 *and *Ccl9 *displayed significant differences, while *Cxcr4 *and *Il18 *had similar levels of expression. Comparing the chemokine gene expression of FBM17.5 to both fetal livers LSK cells, only *Cxcr4 *and *Il18 *showed a significant upregulation in FBM17.5 LSK cells, while only *Ccl4 *expression showed a significant difference between FBM17.5 and FL14.5. Finally, when fetal liver LSK from the different microenvironments were compared, *Ccl4*, *Ccl9 *and *Il18 *(but not *Cxcr4*) had a significant upregulation in the FL17.5 LSK. Taken together, these data show that expression of chemokines and their receptors changes with developmental stage and can be correlated with the microenvironment where the LSK are present.

**Table 3:** Folding expression of chemokines and receptors in LSK across different locations and stages of development

Gene	FL14.5	FL17.5	FBM17.5	Adult BM
Chemokine (C-C motif) Ligands				
*Ccl4*	0.02	0.17	0.18	1
*Ccl5*	0.05	0.2	0.19	1
*Ccl6*	0.02	0.13	0.08	1
*Ccl9*	0.09	0.24	0.18	1
Chemokine (C-X-C motif) Ligands				
*Cxcl2*	0.03	0.24	0.17	1
*Pf4*	0.47	0.31	1.72	1
Chemokine (C-C motif) Receptors				
*Cxcr4*	0.31	0.38	0.76	1
Other Chemokines and Related Genes				
*Cmtm3*	0.72	0.93	0.73	1
*Cmtm6*	0.28	0.22	0.45	1
*Hif1a*	0.5	1.07	0.75	1
*Il16*	0.42	0.37	0.82	1
*Il18*	0.04	0.21	0.57	1
*Mmp2*	0.07	0.2	0.27	1
*Nfkb1*	0.48	0.82	0.84	1
*Tlr4*	0.01	0.11	0.23	1
*Tnfrsf1a*	0.4	0.45	0.71	1
*Tnfsf14*	0.18	0.31	0.43	1
Housekeeping Gene				
*Hprt1*	0.48	0.81	0.63	1

This table demonstrates fold change values for samples in which gene expression was detected at all the points of study, compared to gene expression in adult bone marrow. Adult BM, four-week old adult bone marrow; FBM17.5, fetal bone marrow at Day 17.5 of gestation; FL14.5, fetal liver at Day 14.5 of gestation; FL17.5, fetal liver at Day 17.5 of gestation; LSK, Lineage^- ^Sca-1^+ ^c-Kit^+^.

It is interesting to note that many of the genes included in the Chemokine & Receptors PCR Array showed significant downregulation by volcano plots when the FL14.5 sample was compared to the other three tissues. In the FL14.5 sample, the expression of the majority of these genes was below the limit of detection of the qPCR array. These genes include *Aplnr*, *Bdnf*, *Bmp10*, *Bmp15*, *Bmp6*, *Ccbp2*, *Ccl1*, *Ccl11*, *Ccl12*, *Ccl17*, *Ccl19*, *Ccl2*, *Ccl20*, *Ccl7*, *Ccl8*, *Ccr1*, *Ccr1l1*, *Ccr3*, *Ccr4*, *Ccr5*, *Ccr6*, *Ccr7*, *Ccr8*, *Ccr10*, *Ccrl1*, *Ccrl2*, *Cmtm2a*, *Cmtm4*, *Cmtm5*, *Cmklr1*, *Cxcr7*, *Csf1*, *Csf2*, *Cx3cl1*, *Cxcl1*, *Cxcl10*, *Cxcl11*, *Cxcl12*, *Cxcl13*, *Cxcl15*, *Cxcl5*, *Cxcl9*, *Cxcr3*, *Cxcr5*, *Cxcr6*, *Gdf5*, *Gpr81*, *Il13*, *Il1a*, *Il4*, *Il8ra*, *Inha*, *Inhbb*, *Lif*, *Ltb4r2*, *Myd88*, *Ppbp*, *Rgs3*, *Slit2*, *Tnf*, *Trem1*, *Tymp*, and *Xcl1*.

## Discussion

It has been proposed that the clinical mobilization of hematopoietic stem cells mimics mechanisms that naturally occur during the migration and engraftment of HSC in the fetus and adult [8]. However, the molecular profile of adult and fetal HSC differs significantly. In studies comparing extracellular matrix and adhesion molecule gene expression, LSK from FL14.5 and adult bone marrow differed in more than 20% of the genes studied [34]. This study analyzed only fetal liver LKS cells at 14.5 dpc and not other developmental stages. A more inclusive quantitative analysis, including different fetal developmental stages and microenvironments, is necessary to evaluate differences between the adult and fetal LSK molecular phenotype. Here, we have compared the extracellular matrix, adhesion and chemokine gene expression pattern of LSK populations from FL14.5, FL17.5 as well as FBM17.5 to adult bone marrow LSK in order to identify genes that modulate during development.

We initiated our study by analyzing the frequency and absolute cell numbers of the LSK population as well as LT-HSC in the aforementioned tissues. During ontogeny, the increase in fetal liver HSC numbers takes place after 12.5 dpc, which agrees with our observed HSC increase from 14.5 dpc to 17.5 dpc. However, the number of fetal liver HSCs attains its maximum by 15.5 to 16.5 dpc, reaching a plateau and then declining [8,35,36]. The migration of HSCs to the blood, spleen, and bone marrow by 17.5 dpc [8] could account for the reduction of HSC in the fetal liver at this developmental time, and is consistent with the lack of significant differences observed in the frequencies and absolute cell numbers of LSK and LT-HSCs between the fetal tissues studied. Moreover, when adult bone marrow was analyzed, significant increases were observed in LSK and LT-HSCs cells, suggesting a definitive expansion of the HSCs pool in this tissue.

We sorted the LSK and LT-HSC populations from different locations during development in order to evaluate their molecular profiles. We found that the absolute number of LT-HSCs from these different embryonic sites was very low; especially in the FBM17.5, where an average of five LT-HSCs could be isolated per embryo. This low number of cells precluded us from further analyzing LT-HSC by quantitative PCR array. Therefore, we decided to quantify only the molecular profile of LSK cells, since this population includes LT-HSCs [34]. LSK cells from FL14.5 showed a different gene expression profile than adult BM, with significant differences in 44% of the extracellular matrix and adhesion molecules genes analyzed. This difference is greater than what was previously reported by Ivanova et al. using Affymetrix oligonucleotide arrays [34]. In general, the gene expression of extracellular, adhesion and chemokine molecules in FL14.5 LSK cells was lower than in the rest of the tissues studied. This lower expression could result from the downregulation of migration related molecules at this stage, when LSKs are in a process of vigorous cellular expansion [6]. At later stages of development, no significant differences in gene expression were found between LSK cells from FL17.5 and FBM17.5, or between FBM17.5 cells and adult BM LSK. The lack of difference among these LSK samples suggests a similar extracellular matrix and adhesion molecule genetic program throughout these different microenvironments. Few genes were differentially expressed when the chemokines and chemokine receptor profile was compared in LSK populations at 17.5 dpc and adult BM. Overall, the genetic program in LSK populations was more similar the closer they were in temporal developmental stage. However, there are some genes that remain significantly different between all the stages of development studied and these merit further discussion.

One of these genes is *Col4a1 *(Collagen, type IV, alpha 1), a major constituent of basement membranes [37]. Its expression was significantly different midst LSK populations at all the stages of development, with the exception of FL17.5 and FBM17.5. We hypothesize that LSK cells may contribute to the architecture of its immediate microenvironment by secreting collagen IV onto the extracellular matrix [31]. Matrix metalloproteinase 2 (*Mmp2*) was significantly upregulated in the adult BM when compared with either the FL14.5 or FL17.5 sample. No differences were detected when either FL tissues were compared to FBM17.5, due to its large gene expression variation. MMP2 degrades collagen IV and it could help in LSK motility by releasing them from the extracellular matrix [31]. However, increased expression of *Mmp2 *can mediate also the inactivation of CXCL12, impairing the homing of the HSC to the bone marrow [38]. On the other hand, multipotent progenitors (MPP) have been described to upregulate *Mmp2 *expression in adult bone marrow [31]. Whether our observed increase of *Mmp2 *expression is due to LT-HSCs or MPPs still needs to be clarified. Interestingly, the tissue inhibitor of metalloproteinase 2 (*Timp2*), whose main target is MMP2 [39], showed a pattern of expression similar to *Mmp2*. However, it is also significantly increased in FBM17.5 LSK cells when compared to FL14.5 LSKs. TIMP2 can be upregulated in order to modulate the proteolytic activity of MMP2, which might be required to help LSK cells leave or remodel their microenvironment. Multipotent progenitors downregulate *Timp2 *expression in the adult bone marrow [31] suggesting that its increased expression could reside within the LT-HSCs or ST-HSC populations.

The expression of transforming growth factor beta-induced (*Tgfbi*) increased significantly during development, with no difference between the FL and FBM at 17.5 dpc. TGFBI is a secreted extracellular matrix adaptor protein whose physiological function involves cell-matrix interactions and cell migration. It binds to type I, type II and type IV collagens and it may be involved in endochondral bone formation in cartilage [40]. TGF-β upregulates the expression of *Tgfbi *[41] and its expression is higher on HSC adherent to mesenchymal stem cells [42]. Similarly to *Tgfbi*, the expression of *Sell *(*Selectin L *or *CD62L*) increased at the different stages of development studied without significant difference between the FL14.5 and FBM17.5 sample, due to the large gene expression variability in the FBM17.5 sample. Selectin L is a membrane-bound C-type lectin that binds to cell-surface glycosylated ligands [14] and its role in the migration of hematopoietic stem cells is controversial. *Sell *is highly expressed in mobilized CD34^+ ^cells [43], but it does not appear to contribute to the interaction of HSCs with bone marrow microvessels [44]. Furthermore, our data show that the expression of ectonucleoside triphosphate diphosphohydrolase 1 (*Entpd1 *or *CD39*) increased also at the different developmental stages studied, following a pattern similar to *Sell*. CD39 hydrolyzes NTP and NDP to NMP. Extracellular nucleotides enhance the stimulatory activity of several cytokines and can expand the number of human HSCs repopulating the bone marrow [45]. Taken together, the upregulation of these extracellular matrix and adhesion molecules suggest that they might play a role in the homing and migration of LSK cells between fetal microenvironments during development.

Our results also showed differences in the expression of chemokines and their receptors during development. The expression of the chemokine (C-C motif) ligand 4 (*Ccl4 *or macrophage inflammatory protein-1β, *MIP-1β*) increased significantly in the LSK population from different microenvironments. CCL4 could be involved in migration processes of LSK cells since it has chemoattractant properties and it is inducible in most mature hematopoietic cells [46]. Similarly, chemokine (C-C) motif ligand 9 (*Ccl9*, or macrophage inflammatory protein-1γ *MIP-1γ*), showed a significant upregulation along development in all the tissues studied. It has been shown that rat CCL9 induces the migration of adult bone marrow cells, although the population responsive to CCL9 was not characterized [47]. However, in adult bone marrow LSK, *Ccl4 *and *Ccl9 *expression is upregulated in multipotent progenitors [31]. The expression of Interleukin-18 (Il*18*), a pro-inflammatory cytokine produced by macrophages and other cells, increased also significantly in all the populations studied. The involvement of *Il18 *in hematopoietic stem cell migration is unclear. It will be necessary to discern if the observed increase in the expression of these chemokines at the different microenvironments studied occurs in the MMP or the LT-HSC population.

The expression of the chemokine (C-X-C motif) receptor 4 (*Cxcr4*) augmented significantly in the adult bone marrow when compared to the fetal samples studied. CXCR4 has been described extensively as the key chemokine receptor involved in the migration of HSCs [6,8,21,22,48]. However, few of these studies evaluated the role of CXCR4 in the migration of fetal liver HSCs. Christensen et al. observed that 14.5 dpc fetal liver HSCs did not migrate at the same level as adult HSCs upon stimulation with SDF-1α [8]. The expression of *Cxcr4 *in fetal liver LSK is significantly lower than in adult or fetal bone marrow LSK, which may explain the reduced migration observed in their studies. In summary, the upregulation of the aforementioned chemokines and CXCR4 indicates that they might govern the migration of LSK cells between fetal microenvironments during development.

Surprisingly, adhesion molecules described as relevant for the homing and migration of adult hematopoietic stem cells, such as *Vla4 *(integrin α_4_β_1_) [49] and *Cdh2 *(N-cadherin) [50] did not show significant differences among the populations studied. Similarly, no significant differences were observed with chemokines receptors expressed in adult HSCs, such as *Ccr9 *and *Ccr3 *[22]. However, molecules whose expression does not show significant variance might still play an important role in LSK migration during development.

Our study shows that LSK cells upregulate the expression of several extracellular matrix, adhesion, and chemokine molecules, shown to be components of the bone marrow niche [1]. These data suggest that LSK might contribute to the architecture of their niche [31]. However, cells other than LSK might contribute to a greater extent to the architecture of the stem cell niche, as LSK are extremely rare cells. The final architecture of the stem cell niche might result from the combined molecular and cellular contributions of the stem cells as well as niche cells.

## Conclusions

Our data demonstrate that there is considerable variation in the gene expression of LSK population at different developmental stages and microenvironments. These differences are greater the more temporally distant the populations are. These changes in gene expression should be examined in hematopoietic progenitors derived from embryonic stem cells, which display a genetic profile more characteristic of fetal than adult progenitors, in order to improve their properties for clinical applications such as engraftment. Some molecular candidates to be studied have been identified in this report. Our work expands the understanding of the genetic footprint of LSK cells, opening potential new avenues for research and clinical applications.

## Abbreviations

Adult BM: four-week old adult bone marrow; AGM region: aorta, gonad and mesonephros region; *Ccl4*: chemokine (C-C motif) ligand 4; *Ccl9*: chemokine (C-C motif) ligand 9; *Col4a1*: collagen, type IV, alpha 1; *Cxcr4*: chemokine (C-X-C motif) receptor 4; dpc: days post coitum; *Entpd1*: ectonucleoside triphosphate diphosphohydrolase 1; FBM17.5: fetal bone marrow at day 17.5 of gestation; FL14.5: fetal liver at day 14.5 of gestation; FL17.5: fetal liver at day 17.5 of gestation; HSC: Hematopoietic Stem Cell; IACUC: Institutional Animal Care and Use Committee; Il*18*: Interleukin-18; Lin^-^: mature lineage negative; LSK: Lineage^- ^Sca-1^+ ^c-Kit^+^; LT-HSC: Long Term-Hematopoietic Stem Cells; LTR: long term repopulating activity; *Mmp2*: matrix metallopeptidase 2; MPP: Multipotent Progenitor; NDP: nucleotide diphosphate; NMP: nucleotide monophosphate; NTP: nucleotide triphosphate; pSp: para-aortic splanchnopleura; Sca-1: stem cell antigen-1; *Sdf-1α: s*tromal cell derived factor-1α; G-CSF: Granulocyte-colony-stimulating factor; *Sell*: Selectin, lymphocyte; SLAM: Signaling Lymphocyte Attractant Molecule; ST-HSC: Short Term-Hematopoietic Stem Cells; *Tgfbi*: Transforming growth factor, beta induced; *Timp2*: Tissue inhibitor of metalloproteinase 2.

## Competing interests

The authors declare that they have no competing interests.

## Authors' contributions

JC contributed to conception and design, financial support, collection and assembly of data, data analysis and interpretation and manuscript writing. MGO contributed to conception and design, financial support, data analysis and interpretation, manuscript writing and final approval of the manuscript.
